# Cuticular Lipids as a Cross-Talk among Ants, Plants and Butterflies

**DOI:** 10.3390/ijms17121966

**Published:** 2016-11-24

**Authors:** Francesca Barbero

**Affiliations:** Department of Life Sciences and Systems Biology, University of Turin, Via Accademia Albertina 13, 10123 Turin, Italy; francesca.barbero@unito.it; Tel.: +39-011-670-4538

**Keywords:** ants, plants, butterflies, interaction, chemical signaling, cuticular hydrocarbons, waxes, symbiosis, lipids

## Abstract

Even though insects and plants are distantly related organisms, they developed an integument which is functionally and structurally similar. Besides functioning as a physical barrier to cope with abiotic and biotic stress, this interface, called cuticle, is also a source of chemical signaling. Crucial compounds with this respect are surface lipids and especially cuticular hydrocarbons (CHCs). This review is focused on the role of CHCs in fostering multilevel relationships among ants, plants and Lepidoptera (primarily butterflies). Indeed, particular traits of ants as eusocial organisms allowed the evolution and the maintenance of a variety of associations with both plants and animals. Basic concepts of myrmecophilous interactions and chemical deception strategies together with chemical composition, biosynthetic pathways and functions of CHCs as molecular cues of multitrophic systems are provided. Finally, the need to adopt a multidisciplinary and comprehensive approach in the survey of complex models is discussed.

## 1. Introduction and Outlines

The evolutionary success of organisms relies on their adaptability to various environmental conditions. The integument represents the outer, functional and structured interface between an organism and its habitat [1,2]. It functions as a physical barrier to pathogens or predators, provides protection against wounding and injuries [3,4], regulates the exchanges of water, primarily, but also plays a role in respiration, determination of inner temperature, body movements [5,6,7], and intra- and interspecific communications or sensing [1,2,8].

**Figure 1:**
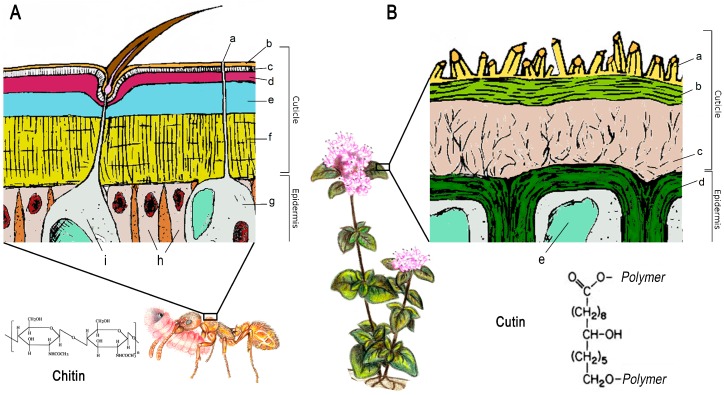
Cuticle scheme of: (**A**) insects; and (**B**) plants. (**A**) a, glandular duct; b, cement; c, wax; d, epicuticle; e, exocuticle; f, endocuticle; g, glandular cell; and h, epidermal cell with nucleus; i, trichogen cell (**B**) a, epicuticular waxes; b, proper cuticle; c, pectine cuticle layer; d, cell wall; and e, vacuole. Chemical structure of insect chitin and plant cutin is also reported.

Curiously, and despite being phylogenetically unrelated, plants and arthropods share a very similar external tegument, which, in both cases, is called “cuticle” and is made of a stratified deposits of organic compounds on the epidermal cells (Figure 1). The challenging need to maintain the water balance in aerial habitats could explain this remarkable case of parallel evolution [1]. In both taxa, the primary function of the cuticle is the prevention of water loss, a feature that is mainly determined by the physical arrangement of surface lipids [6,7,9,10]. More in general, terrestrial organisms have to cope with a “changing” environment, where temperatures, humidity and sun radiations are not constant. Thanks to the cuticle, acting as a plastic barrier able to respond to outer variations by modulating its characteristics (not exclusively its waterproofing), some plants and arthropods survive also in extreme conditions [7,9,11].

The cuticular chemical variability found among species, but also in distinct part of the same insect or plant, suggests a wide range of ecological functions played by this barrier [12]. This review explores the role of the cuticle, acting as a source of chemical signaling, in fostering multilevel relationships between plants and insects. Pivotal elements of the cuticle with this respect are undoubtedly its surface lipids and especially the long-chained, saturated, unsaturated, and branched hydrocarbons. Cuticular hydrocarbons (CHCs) have been intensely investigated in social insects as they are known to be involved in nestmate recognition [13], whereas in plants their main function is in the limitation of water loss [14], as a barrier to plant pathogens [4] or as taxonomical markers [15]. In contrast, there is a lack of information about the CHC contribution as communication signals or cues in multitrophic interactions. CHCs are multifarious mediators in plant–insect associations (see Section 5), which can be secondarily exploited by eavesdroppers (see Section 6). However, the evaluation of benefits for the emitter or the receiver (or both) is far from being straightforward or conclusive, therefore here CHCs are treated generically as stimuli, irrespectively of whether they are true signals or cues [16].

Since symbioses between insects and plants may concern several distantly related taxa, this survey is narrowed to systems involving ants, plants and Lepidoptera as: (i) ideal models to study co-evolutionary dynamics; and (ii) taxa for which bulk of eco-ethological, molecular and physiological information exists.

After concisely describing myrmecophilous interactions, a synthesis beyond the current state of the literature on chemical composition, biosynthetic pathways and functions of CHCs as molecular signals in these multitrophic systems is provided. Finally, the opportunity to follow a comprehensive approach in the inspection of multiple level associations is discussed, suggesting new pathways for future research.

### Why Ants?

Among insects, ants are one of the richest animal groups in terms of abundance, biomass, and diversity. The evolutionary success of ants is likely due to their social organization enhanced by a complex communication system, as well as to their morphology, physiology and ecology [17]. In several terrestrial ecosystems, a considerable amount of individuals, coordinated by means of chemical cues in collective-decision making, represents a key factor for their ecological dominance [18]. Ants lack in a hierarchical control, but their structured labor system as well as the colony plasticity to respond to internal and external environmental variations, by regulating the number of workers involved in distinct tasks, have proven to be a rewarding strategy [19,20].

Ants also show a wide geographical range, they are found in virtually all existing lands, with a few exceptions such as Iceland, Greenland and the Antarctic region. They are adapted to colonize even extreme habitats, and deeply shape the environment in which they live, by moving soils or conveying energy and cycles of nutrients [21]. In addition, ants often represent the main predators of other insects or small invertebrates, the primary seed-disperse organisms or even the major herbivores of their ecosystem. They do not merely defend their nest, but also the space surrounding their colonies [17].

Parallel to ant rise, a number of complex symbioses, sometimes with distantly-related organisms, have occurred. Several associations have favored the ant evolutionary success, e.g., those with fungi [22] or trophobionts [23]. Conversely, the colony resources or the enemy-free space provided by patrolling ants have attracted many social parasites that heavily impact the ant society fitness [24]. It is uncertain when these associations exactly arose, although there is evidence that they are evolutionarily ancient phenomena [25]. Peculiar traits of ants as eusocial organisms therefore allowed the evolution and the maintenance of a variety of associations with both plants and animals, called myrmecophilous (ant-loving) organisms [17,26,27,28] or in the case of obligate plant symbioses, myrmecophytes [29]. These interactions are cursorily described in the following section.

## 2. Myrmecophilous Interactions

The proliferation and forest colonization by angiosperm between 60 and 100 Ma are considered one of the driving forces shaping the diversification of several herbivore lineages, including ants [30]. Angiosperms and their litter primarily offer ants a complex and more diverse habitat to be colonized [31], while the herbivores associated might represent a source of food, directly as prey or incidentally providing ants with secretions or honeydew [23]. Plants gain benefits from ant patrolling and catching herbivore preys to such an extent that they evolved morpho-physiological modifications to enhance ant occurrence or visiting. A seminal example is provided by the *Acacia*–*Pseudomyrmex* association, where plants use all possible adaptations, such as secretory structures, extrafloral nectaries (EFNs), food bodies, and specialized *domatia*, to attract ants as bodyguards against herbivores or, in the specific case, to deter encroaching vegetation [32]. A plethora of different associations between plants and ants exists, ranging from facultative to obligate and having positive (pollination, seed dispersal, and direct or indirect protection against biotic damage) but also neutral or negative effects on plant fitness (for a detail description of these interaction, please refer to reference [29]).

How can the presence of ants be disadvantageous for plants? Besides straightforward effects of ant species that directly feed or damage plant tissue, such as the well-known leaf cutting ants [17], indirect detrimental impacts are also known to occur (e.g., [33]). For instance, the mutualism involving *Duroia hirsuta* plants and *Myrmelachista schumanni* ants shows associated costs [34]. These ants defend and create monospecific gardens (called devil’s garden) of their host plant, *D. hirsuta*, by poisoning other plants in the surroundings with formic acid [35]. In this way, *M. schumanni* enhances the growth of its own nesting places, with a positive effect also for the host plants, but simultaneously provides a bulk of new attractive resources for herbivores. Thus, the herbivore pressure becomes higher inside than outside devil’s gardens. *M. schumanni* ants are not able to counterbalance the increase of herbivores and prove to be less effective in counteracting their attacks against *D. hirsuta* [34]. The overall balance for the plant can also be negative if the impact exerted by sap-feeding hemipterans or lepidopteran larvae, feeding and injuring the plants as they are protected by ants from their predator or parasitoids, outweighs the benefits of ant protection against other herbivores [36,37]. These profiting aphids and caterpillars have evolved chemical strategies to “cheat” ant detection or to attract ants with food rewards (see Section 6). The latter organisms are called trophobionts as they provide liquid food for ants, in the same way as EFNs do.

DeVries [38] suggests that this multifaceted scenario, involving host plants, “harvesting” ants (i.e., their diet relies on liquid secretion) and natural enemies, could have favored the fostering of butterfly–ant symbiosis. While most plant–ant interactions have been regarded from the plant point of view (adaptation, chemical signaling, and positive or negative balance are mostly referred to the plant), butterfly–ant associations are primarily based on the impact they produce on ants. Myrmecophiles belong to various insect orders (e.g., Hymenoptera, Diptera, and Coleoptera), but one of the most investigated taxon is undoubtedly Lepidoptera, primarily butterflies, whose interactions with ants have been broadly reviewed [37,39,40]. As it occurs in plant associations, the majority of butterfly myrmecophilous interactions are facultative and unspecialized [37,41]. However, in the same way as plant *domatia* provide a protected nesting place for ants, ant colonies themselves are safe refuges, climatic stable environment and constant source of food for those ant-sized organisms able to overcome nest defenses [26,42]. Generic adaptations such as armor or food secretion are usually enough to foster facultative or unspecific interaction, enabling myrmecophiles to access the enemy free-space provided by ants. In contrast, more intimate relationships, where the intruders used to live inside the colonies to exploit nest resources, require either “stealth” or the ability to corrupt honest communication signals of ants [24,39].

Hence, mechanisms and adaptations evolved by butterfly immature stages to interact with ants depend on the degree of association they show and could aim at: (i) pacifying or confusing ants, e.g., secretion of rewarding droplets from dorsal nectary organs (DNOs) or emission of allomones by tentacle organs (TOs) to appease or alarm ants [43]; (ii) becoming more resistant to ant attack evolving morphological modifications, e.g., thickening of cuticles [44]; or (iii) breaking the communication codes of the host, e.g., chemical or acoustical signaling [45,46,47]. Morphological, behavioral, chemical or acoustical adaptations of myrmecophile organisms have been recently reviewed [24,28,48,49].

## 3. Cuticular Lipids of Ants: Structure, Function, and Biosynthesis

The ant ability to coordinate efficiently the actions of hundreds of nestmates relies on multimodal, chemical [49,50] tactile [17], visual [51] and vibro-acoustical [47,48,52,53,54] communication.

Even though a consistent body of evidence is increasingly recognizing vibrations a pivotal role [47,48,52,53,54], semiochemicals remain the primary means of ant communication. Chemical signals cause and enhance a broad array of behaviors and functions, encompassing alarm, recruitment or trail cues, necrophoresis (i.e., the carrying of dead nestmate bodies) or trophallaxis (i.e., food exchange) activities and have been extensively reviewed (e.g., [49,55]).

*Nestmate recognition.* The kin recognition is the basic mechanism underpinning ant social cohesion and ensuring that altruistic behavior is addressed towards the correct and related receivers [56]. The ability to recognize other individuals as nestmates is achieved through the exchange of chemical signals. Cuticular hydrocarbons (CHCs) have long been assumed to play a major role with this respect ([57] and references therein). These long-chain hydrocarbons (linear and branched alkanes and alkenes) cover the external layer of the cuticles and, beyond their communicative role, they mainly serve to avoid desiccation and protect insects against biotic and abiotic stress [57].

In ants, CHCs are extremely diverse; collecting data on 78 ant species, Martin and Drijfhout [58] found a total of about 1000 different CHCs occurring in peculiar, species-specific mixtures, irrespectively of species phylogeny. Most abundant are *n*-alkanes followed by monomethylalkanes, dimethylalkanes, alkenes, dienes and, more rarely, trimethylalkanes. Methylalkenes, methylalkadienes, trienes and tetramethylalkanes are seldom produced by very few ant species [58] (Table 1). Long-chain compounds are commonly thought to increase waterproofing ability of the cuticle [7], whilst blends of methyl-branched and unsaturated compounds generally decrease the melting temperature, thus reducing the desiccations resistance [59]. An example of this is provided by *Pogonomyrmex* forager ants, which are heavily exposed to desert. Foragers prevent the water loss by possessing a disproportionate higher rate of alkanes with respect to other colony members, living inside the nests [60].

In addition to CHCs, other classes of compounds were found in the cuticle of ants, but because they do not show a role in mediating recognition between nestmates [60,61,62,63], they have been often disregarded or removed on purpose prior to extraction [61]. Lockey [2] suggested that a minor component of surface lipids encompasses traces of fatty acids, alcohols, esters, glycerides, sterols, aldehydes, and ketones (Table 2). Wax esters and esters of fatty acids with fatty alcohols are the most commonly found [61,62].

**Figure 2:**
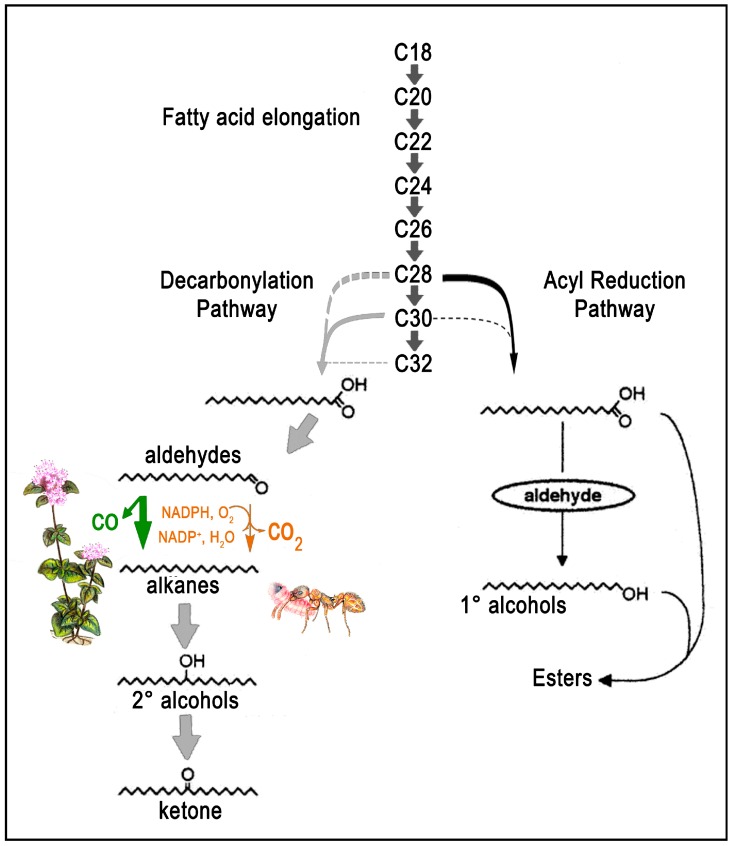
Biosynthetic pathway of cuticle compounds, modified from [69]. Decarbonylation (grey arrows) and acyl reduction (black arrows) pathways are shown. Dotted arrows indicate potential, minor steps. Plant and insect (adapted from [67]) decarbonylation are marked in green and brown, respectively. See the main text for further explanation.

Ant CHC biosynthesis (Figure 2) is still a controversial issue. In contrast to plants, where acyl-CoA is converted to an aldehyde which is then decarbonylated in absence of oxygen, with the release of carbon monoxide [64], in insects the decarboxylation leads to hydrocarbon and carbon dioxide and requires molecular oxygen and the presence of a NADPH-dependent cytochrome P450 enzyme [65,66]. The correct length of the carbon chain is thus achieved by the reduction/elongation of fatty acyl CoA precursors in all three classes of CHCs [67], but the insertion of methyl substituents in branched alkanes requires a methylmalonyl-CoA, whilst alkanes and alkenes are produced by malonyl-CoA. A desaturase finally determines the positions of double-bonds in alkenes [68] (Figure 2).

No matter the mechanism of biosynthesis, CHCs are synthesized in the oenocytes, which are cells associated to fat bodies or to the epidermis, and afterwards conveyed to target tissues by lipid carriers (apparently lipophorin) through the hemolymph [70]. Chemical profiles are genetically determined [8], but the qualitative variation of cuticular compounds is likely to be due to gene expression regulation rather than to gene loss [68]. In addition, as the biosynthesis of certain compound classes is linked to the exogenous uptake of peculiar amino acids, the diet might also influence and cause variations in epicuticular chemical profiles of insects [71].

During trophallaxis (CHCs can be stored in the post-pharyngeal gland) or grooming activities, characteristic and specific mixtures of linear alkanes, branched alkanes and alkenes are shared by individuals living in the same nest. CHCs, although mostly not volatile, are thought to contribute to the creation of a qualitatively similar “colony odor” (also known as “*gestalt*”) thus enabling ants to discriminate between nestmates and intruders [72]. Further variation in CHC patterns identifies differences in sex, caste, developmental stage or task status [73,74,75].

Various classes of CHCs play a different role in ant recognition skills. Branched alkanes and alkenes can be discerned based on their methyl group or double bond position, whilst the discriminant power of linear alkanes only resides in the chain length, therefore they seem less important [76,77]. Furthermore, alkanes are less reliable candidates for having a crucial function in nestmate recognition, because they are ubiquitous [58] as, in combination with monomethylalkanes, significantly concur to basic, structural functions of the cuticle (i.e., waterproofing adjustable to various environmental conditions) [59]. However, some ant species uses alkanes for signaling task [75,78] or mating status [79]. Conversely, dimethylalkanes, which are species-specific and in some cases colony-specific, have been suggested by several authors as important cues in recognition process ([58] and references therein, [80]). Nevertheless, Greene and Gordon [81] showed that a mixture of at least two CHC classes is fundamental to elicit a recognition response. To assess the identity, each member of the colony inspects by antennae the hydrocarbon patterns of other workers and compares it to its own [82], which therefore functions as a template [13,83,84]. Nestmates are then disregarded, whereas the encounter with an intruder or a conspecific causes an antagonistic behavior [85,86].

There is a paucity of studies directly assessing how CHCs are perceived. Antennal sensilla receive the dendrites of several Olfactory Receptor Neurons (ORNs) which, in cooperation with their molecular receptors, are thought to be involved in chemical signal detection and transduction (e.g., [82,87]). The role of specialized sensory hairs, namely sensilla basiconica, in detecting ant CHCs has been investigated in *Camponotus* ants [82,87,88,89]. A seminal electrophysiological experiment was performed by Ozaki and collaborators [82] who described an antennal sensillum responding specifically to mixtures of non-nestmate CHCs, but insensitive to nestmate blends. Because responses were also elicited by antennae isolated from the ant head, authors argued that a sensory filter acted peripherally as a template. A sensory adaptation to a continuously present stimulus (i.e., the nestmate CHCs) has been suggested to occur, thus only cues about non-nestmate odors are carried to the brain [82]. Nevertheless, if nestmates are not detected, this hypothesis fails to explain how ants are able to recognize distinct castes or tasks within the colony [73,75]. Moreover, the colony odor is unstable in time and influenced by internal (e.g., age, caste, and reproductive status) as well as environmental factors (e.g., [90,91]). Therefore, the colony template formed in the neurons should be incessantly updated, following a learning process termed “template reformation” [89]. By coupling electroantennography and calcium imaging, Brandstaetter and colleagues [87,89] found that *Camponotus* ants are not anosmic to nestmate CHCs, as previously suggested [82]. Instead both member and non-member odors elicited a neuronal response [87]. The activity patterns measured in antennal lobes were consistent with the segregation of odor perception process in different anatomical regions [89]. These parallel processing could provide a quicker and more efficient mechanism to discern complex stimuli such as the colony odor, but much with this respect remains to be investigated and unraveled [89]. Very recently, Sharma and colleagues [88] using finer electrophysiological and behavioral bioassays revealed that basiconic sensilla are indeed broad-spectrum detectors for hydrocarbons of both nestmates and non-nestmates, as well as of various ant castes, and that they activate the antenna causing a behavioral response. Moreover, the capacity of such sensors in discriminating *R* and *S* enantiomers has been highlighted. This ability of insect to detect even small molecular differences, such as the presence of unsaturation or methyl groups of the same chain-length has also been assessed by other research groups [92,93], though the exact molecular basis of nestmate perception is still controversial. Guerrieri et al. [80] showed that the addition of a given compound (or the increase of its concentration), instead of the lack of some compounds, is able to elicit an ant attack, thus indicating that the comparison is based on inclusiveness rather than on broad odor similarity.

Overall, nestmate recognition is far from being a perfect or all-or-nothing mechanism, because the colony odor is seldom a consistent and homogeneous sum of all individual chemical profiles. The range of variation in the *gestalt* odor could be influenced by the colony size, i.e., in small colonies allogrooming is favored and homogeneity ensured, or by the degree of polygyny (numbers of queens) which causes genetic and, consequently, odor heterogeneity. Therefore, dynamic, contest-dependent thresholds of similarity at which members and non-members differ exist [94]. This enables individuals to avoid rejecting self-colony members by mistake.

The margin of errors in nestmate recognition process is one of the weakest points of ant defense strategies. These cues are exploited by “invading” organisms, which mimic ant CHCs to be mistaken as colony members or ignored (see Section 6).

## 4. Comparison between Plant and Ant Cuticular Lipids

The development of a waterproof barrier is also one of the most important adaptations in the evolution of plants. The plant cuticle has allowed the successful colonization of terrestrial habitat about 400 million years ago [9].

A detail review of the structural and chemical compositions of plant cuticle has been recently published by Fernandez et al. [95]. Here I focus on essential differences with respect to ant’s cuticle and on basic concepts useful in understanding its role in biotic interactions.

The plant cuticle is made of two main constituents, i.e., the cuticular waxes and a polymeric insoluble organic component, the cutin [96] (Figure 1). Ants and plants share a quite similar wax layer, consisting in blends of very-long-chain aliphatic hydrocarbons, which can be variously substituted broadening the chemical profile with the presence of primary and secondary alcohols, ketones and diketons, esters, aldehydes and fatty acids [97] (Table 2).

In plants exclusively, the presence of secondary metabolites such as flavonoids and triterpenoids is also documented [15]. The main difference between insect and plant cuticle is found in the composition of the polymeric component. Even if doubts about the molecular structure of cuticle persist (see [95]), one widely accepted model suggests that, in plants, waxes are enclosed (intracuticular) and superimposed (epicuticular) in a cutin matrix, made of glycerol and ω- and mid-chain hydroxyl and epoxy C_16_ and C_18_ fatty acids [15], whilst the polymeric network of insects consists of poly-*N*-acetylglucosamine, namely the chitin, and proteins [2] (Figure 1).

A direct causal relationship between each plant cuticle component and its function is still unclear, but authors generally agree in ascribing the multifunctional nature of the cuticle to its multifaceted mechanical and chemical structure [99]. This wax heterogeneity is also observed among plant species or genotypes, as well as between organs belonging to the same individual or even within ontogeny phases, and can be influenced by physiological or environmental conditions (e.g., [95,100,101,102]). Despite this variation, some wax pattern or even single compound (e.g., Δ-6 fatty acids in the Ranunculaceae [103]) are found to be typical of certain plant groups, to such an extent that epicuticular compounds have been successfully used in plant chemotaxonomy of different families including angiosperms [104,105,106,107] and gymnosperms [14].

## 5. Role of Plant Surface in Biotic Interactions as Sensing and Signaling Source

The cuticle is the first site of interaction between an insect and the plant used for both nutritional and reproductive scopes. Here, initial chemical cues influencing insect development or behavior are harbored, because plants would benefit of preventing biting or oviposition before mechanical damages take place [108]. Although secondary metabolites, mostly volatile compounds, have received the highest attention as allelochemicals or attractants in multitrophic interactions, they may also be adsorbed on the epicuticular waxes and to a certain extent may work as short-range signals synergistically with cues provided by cuticular lipids [109,110,111,112].

Nevertheless, there is evidence that long-chain alkanes and alcohols can act as cues for host plant selection (e.g., [113,114,115,116]) or deterrence [117]. The addition of linear alkane blends to homogenates of host plants enhances the oviposition by diamondback moth, *Plutella xylostella* [110], and larval survival is higher on waxy plants than on plants from which lipids have been artificially removed [118]. Moreover, mixtures of cuticular lipids applied to cabbage leaves elicit distinct behavioral responses, concurring to demonstrate an allelochemical activity of cuticular lipids in driving host selection by this moth [119]. Eigenbrode and Espelie [12] report examples of the contribution of epicuticular compounds in hindering the feeding of some herbivores and of their direct toxicity for insects after ingestion [120]. Even though the majority of these studies are rather correlative, they show that epicuticular extracts inhibit or reduce the larval growth of several species of Lepidoptera, such as *Spodoptera frugiperda* or *Helicoverpa zea* [121,122].

Analogously to plant benefits, herbivores too take advantage of quickly assessing the host plant quality (e.g., [123,124]). Given that quali-quantative changes in epicuticular waxes have been documented in various plant organs or according to different senescence status, their composition can act as a proxy for the specialist herbivore to identify the “ideal” oviposition or feeding site [125]. For instance, distinct waxes showed a dissimilar degree of deterrence on harvesting by leaf-cutting ants [126,127] as a secondary outcome from selection of plant resistance to fungal disease [128]. The forager’s choice of a proper leaf to cut is crucial for the successful rearing of ant symbiotic fungus, because hydrophobic characteristics and toughness of the substrate could limit the hyphae growth or penetration, respectively. However, as these two features did not differ significantly among the plant species tested, Sugayama and Salatino [126] argued that the ant ability to discriminate suitable leaves resided in their perception and selection of a proper epicuticular chemical profile.

Besides, wax chemical composition also influences the three dimensional crystal structure of surface lipids, consequently affecting the physical properties of the cuticle. The latter include “slipperiness” and reflections, which can be both involved in plant–insect interactions. While variations in the reflection index, hence in the pattern of colors perceived, may affect the behavior of insects using visual cues for host selection, slippery properties are directly involved in ant–plant interaction [125]. The cuticle microstructure of *Macaranga* plants, for instance, prevents the majority of walking insects to climb its stems and only symbiotic ants (*Crematogaster* and *Camponotus* spp.) show specific adhering abilities, adaptive to rise on and to exploit a competitor-free space [129]. In this specific case, triterpenoids are correlated with the presence of selective waxy barriers and are therefore thought to be involved in the formation of wax blooms of *Macaranga* plants [130]. It is worth noting that the same waxy slippery surface strategy is employed by carnivorous pitcher plants (e.g., genus *Nepenthes*) to catch and trap insects as source of nutrients [131], whereas sticky cuticles deter herbivores in the case of the Inuleae [104].

Finally, changes in epicuticular waxes induced by oviposition events may function as synomones for the plants, because they could be detected by egg parasitoids during host finding. Clear evidence is reported by the elegant experiment of Blenn and colleagues [132], who found that the egg-laying of *Pieris brassicae* butterflies on *Arabidopsis thaliana* elicited wax modifications, with increasing tetratriacontanoic acid (C34) and decreasing tetracosanoic acid (C24) contents: the latter serving as attractant for the egg parasitoid, *Trichogramma brassicae*.

## 6. Lepidoptera Eavesdropping on Ant-Plant Cross-Talk: CHC Crypsis, Camouflage and Mimicry

Organisms willing to live undetected or to exploit resources of ant–plant symbioses have to subvert communication signals exchanged in these interactions. Instances of butterfly and moth caterpillars simulating plant or ant CHCs to take part in multitrophic systems are discussed after a brief overview on specific terminology.

True “mimicry” means that the entity is misidentified, but detected and treated as a specific object. The term “crypsis” instead is referred to the ability of the entity to match the environment, therefore items are generally unidentified and ignored [133].

Myrmecophiles use various strategies termed as:

- Chemical insignificance, by reducing their surface compounds [134,135];
- Chemical camouflage, by passively acquiring the cuticular profile of host from contact or diet [49,136,137];
- Chemical mimicry, by the active biosynthesis of mimetic compounds [49,136,137].

Examples of crypsis obtained through camouflage, where herbivores passively (primarily through diet) obtain an epicuticular profile similar to that of their host plants, are reported by Eigenbrode and Espelie [12]. Nevertheless, in plant–herbivore interactions, there is evidence of crypsis by mimicry too. The mimetic chemical profile showed by codling moths, for example, is not achieved through food intake, because also individuals fed with artificial diet are able to actively synthesize compounds peculiar of their host plant fruits. In this system, further trophic levels (i.e., codling moth egg parasitoid and its hyperparasite) share strong similarities in the surface chemistry [138].

When the association is not specific, and a single plant is visited by several ant species, the best strategy for an herbivore, willing to exploit its host without being attacked, is to resemble the background. The matching between host plant and *Biston robustum* (Lepidoptera: Geometridae) larval CHCs has been proved to be an efficient chemical adaptation to escape raid by various ant species [139]. However, larvae growing on a host plant and then transferred on a distinct plant species lose they crypsis and are attacked by resident ants, at least until their molting. Authors observed that the feeding on the new plant is necessary to provide the larvae with the novel set of mimetic CHCs. Therefore through food intake these moths are able to match several backgrounds and deter the associated ants [139]. A similar strategy is reported for *Mechanitis polymnia* (Lepidoptera: Nymphalidae) larvae, which are able to elude *Camponotus* ants by mimicking the CHC profile of their host plants, *Solanum tabacifolium*, but are attacked when moved to non-host plants [140]. Unlike to the previous study, Portugal and Trigo [140] did not analyze and compare CHCs involved in the crypsis, and reached their conclusion on the basis of transplant/behavioral experiments. These results are indeed clear-cut, but the comparison of chemical profiles would have provided fundamental clues about the coevolution of this symbiosis (see Section 7). Similarly, Whitehead and colleagues [141] studied a system where a specialist herbivore, *Piezogaster reclusus* (Hemiptera: Coreidae), uses its CHCs to feed undisturbed on acacias (*Vachellia collinsii*) defended by *Pseudomyrmex spinicola* ants. Authors found that cuticular compounds are necessary for *P. reclusus* to prevent attack from ants, but they do not protect the herbivore if transplanted on other *P. spinicola*–*V. collinsiii* associations. This suggests that the cuticular profile of *P. reclusus* is colony-specific or host plants specific, but again this remain an unresolved question, because the chemical pattern was not analyzed [141].

Strategies of CHC chemical deception were recently elucidated by Inui and colleagues [142] in a multilevel system involving *Macaranga* myrmecophytes, their species-specific symbiont ants (*Crematogaster* spp.) and specialist herbivores of the genus *Arhopala* (Lepidoptera: Lycaenidae). Ants are strictly linked to their *Macaranga* host plants as nourishment suppliers in terms of food bodies as well as of honeydews from plants’ symbiont coccids. Ants obligately patrol leaves and their aggressiveness towards enemies can be enhanced by specific volatiles emitted by the damaged plant parts after herbivore wounding [143]. Nonetheless, *Arhopala* caterpillars are able to live undisturbed on these ant-associated plants, thus feeding and developing in a well-protected, enemy-free space. The mechanism allowing the butterfly larvae to deceive ants is species-specific and varies from deception to chemical insignificance [142]. Unlike cases reported earlier, larvae of *Arhopala dajagaka* are found to exhibit CHC profiles closely matching those of the ants inhabiting their host plants (*Macaranga rufescens*), instead of those possessed by the plants themselves. Whatever is the mechanisms enabling the chemical deception (camouflage or mimicry), the CHC profile of *A. dajagaka* is actively recognized as a nestmate signature by ants of the host plants and as an “intruder cue” by ants of non-host plants. Therefore, these lycaenids are fully accepted in ant symbiosis involving *M. rufescens*, whilst they are attacked by ants colonizing other *Macaranga* species and showing distinct chemical signatures. In a second species, *Arhopala amphimuta*, the presence of CHCs peculiar of caterpillars (and which do not occur on ant associated to the host plants *Macaranga trachyphylla*) “tags” the lycaenid larvae as strangers. Hence, these invading organisms should rely on other strategies to elude attacks of ants. Authors suggested that sugar secretion from *A. amphimuta* DNO could have a role in appeasing symbiotic ants of *M. trachyphylla.* Larvae of *Arhopala zylda* have evolved a third strategy, which can be ascribed to the chemical insignificance type. *A. zylda* larval surface carries only traces of seven CHCs, less than one third of the number forming the chemical profile of the other two *Arhopala* species, and caterpillars are generally ignored by both ants inhabiting host (*Macaranga beccariana*) or non-host plants. Unfortunately *Macaranga* cuticular lipids were not taken into account in this study, whereas a comparison of both larval and ant chemical profiles with that of the specific host plant would have helped in understanding the patterns observed. Clues suggesting a role of *Macaranga* with this respect are provided by the fact that triterpenoids, which are typically found in plant cuticle, are unexpectedly detected in *A. zylda* epicuticular blends.

Among lycaenids, the most studied are social parasite butterflies of the genera *Niphanda* and *Maculinea* [24,144]. These butterflies spend 9–22 months inside the host ant nest, where caterpillars are reared on regurgitation by attendance workers (some *Maculinea* species called “predatory” feed directly on the ant offspring). At III or IV instars, after feeding on aphids tended by the host ants (*Niphanda fusca*) or after a two-week phytophagous life on a specie-specific food plant (*Maculinea*), larvae of both butterflies do not actively enter host ant colonies, but are carried and adopted by foraging workers, *Camponotus japonicas* and *Myrmica* spp. respectively, thanks to their ability to mimic ant brood CHC profile (*Niphanda fusca* [145]; *Maculinea* spp. [45,46,146,147,148,149,150]). Once inside the host colony the epicuticular composition of *N. fusca* larvae gets a closer match of male ant profile by acquiring, through camouflage, specific blends of CHCs. Is by pretending to be ant males, that larvae of the butterfly parasite obtain care and food for their whole stay in host nests [145]. A better matching of the profile of its host ants is also achieved by *Maculinea* larvae, even though this is not devoted towards a high status ant caste, as in the case of *N. fusca*. The strategy used by *Maculinea rebeli* larvae to get fully integrated in their host colony is a true example of chemical mimicry, as parasite larvae showed the ability to actively synthesize CHCs of their primary host ants [45,46].

Although the *Maculinea–Myrmica*–host plants system is a well-studied model, the role of individual CHC, their origin, their link with the larval food plant together with numerous other molecular, physiological, biochemical, etho-ecological issues have yet to be disentangled.

## 7. Conclusions

Some of the evidence elucidating ant–plant–herbivore interactions from behavioral, evolutionary, chemical, or molecular perspectives is reviewed here. Although our knowledge of mechanisms underpinning these interactions is rudimentary, a plausible hypothesis could be that epicuticular lipids play a pivotal role in fostering such a complex association. It is increasingly clear that beyond their function of preventing the water loss [1], CHCs are also used as cues or communication signals in these symbioses (see Section 5 and Section 6). Thus the evolutionary convergence of plant and insect cuticle, which can be primarily attributed to the need of facing the external environment, could have brought secondary, but even more important ecological consequences. It has already been demonstrated that ant and plant evolutionary pathways are strictly linked [30] and that their interaction is also at the basis of the evolution of ant–butterflies symbiosis [38]. It is remarkable that the success of most of these associations, at least pairwise, is mediated by cuticular compounds (see Section 5 and Section 6). The latter are indeed the main stimuli through which ant perceived their world (see Section 3), not only nestmates or intruders, but more in general the organisms to which they interact, being them mutualistic butterflies or host plants.

The information carried by CHCs has a multifarious, not fully unraveled nature influencing insect behaviors chemically, physically, as blends or maybe through single compounds, to such an extent that is comparable to the variety of functions conveyed by volatile compounds (e.g., plant secondary metabolites, insect pheromones) on which research has been mainly focused. Cuticular lipids can affect interactions directly, acting as allomones, attractants for feeding, nesting or egg-laying as well as toxic or repellent molecules, or indirectly as kairomones or synomones. The caterpillar eavesdropping on ant–plant communication deeply relies on CHC deceptions. Unfortunately, most of the evidence available so far is only correlative and the majority of studies described here took into account pairwise, bi-level interactions independently. It is almost unbelievable, for instance, that even in a very well-known model such as that involving *Myrmica* ants, *Maculinea* butterflies and their larval host plants (LHP), the CHC profiles of the LHPs have never been assayed or taken into consideration. However, these are obligate and highly specialized interactions and the comparisons between plant and larva CHCs could shed light on basic mechanisms underlying their co-evolution. Why do these butterflies only feed and develop on peculiar plant species? Are plants the source of ant mimetic CHCs used by caterpillars in the following adoption process? Is this the reason for the evolution of monophagy in the *Maculinea* genus? Does egg-laying by a butterfly female change the surface lipids thus signaling another adult that the plant has already been occupied? Alternatively, is this the near-field cue used by butterfly parasitoids to locate their hosts on the LHP? Do ants use CHCs to discriminate LHPs? These are only few examples of questions that could be answered or inferred by evaluating also plant CHCs, in addition to ant and butterfly cuticular compounds.

Filling this gap in the knowledge of fundamental processes occurring in ant–plant symbiosis could provide new insights for the comprehension of several biological models. Unfortunately, the lacking of a community context approach in the study of multitrophic interactions is common and concerns also research not necessarily linked to cuticular lipids. In general, this has concurred to underestimate the indirect cues provided by the “information web” (e.g., CHCs), which covers and is interwoven to the “food web” [151]. There are a few examples of studies considering all possible relationships, energy fluxes, exchange of signals occurring among all the organisms involved in multitrophic interactions (e.g., [152,153]), but these are almost absent in the case of surveys involving ant–butterfly–plant systems (but see [154]). It is indeed necessary to take into account the overall variation of associated costs and benefits of these systems to fully comprehend their co-evolutionary dynamics and the whole array of possible outcomes ([151,155] and references therein).

Using a comprehensive, community context-dependent approach in the survey of complex systems is timely and feasible, due to the available technology, the short-time analysis required, and the existence of active and multidisciplinary research networks. Cuticular lipids provide a good model to disentangle interacting selective pressures in complex systems. Further research, beyond involving plant and animal physiologists, entomologists and botanists, behavioral and chemical ecologists to assure a wide range of perspectives, should address the investigation of: (i) CHC perception and complex odor discrimination; (ii) ways of how CHC are acquired from the background, through the diet or the contact with other organisms as well as by the active synthesis of new compounds; (iii) roles played by classes, blends or single compound in defense, attraction or mimetic purposes; and (iv) genetic bases and gene expression regulation of CHC variations, not only in insects but also in plants as induced response by biotic stress.

Although several questions remains unsolved, aspects reviewed here concur to support the idea that cuticular lipids are key factors for the survival and the success of multiple interactions involving ant, plant, herbivores and predators.

## Figures and Tables

**Table 1:** Chemical structure and occurrence of the most representative CHCs of ants. The percentage of occurrence is adapted from [58].

Compound Classes	Chemical Structure	Occurrence in Ant Species (%)
*n*-Alkanes	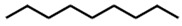	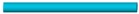
Monomethylalkanes	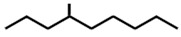	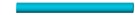
Dimethylalkanes	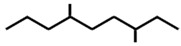	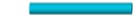
Alkenes	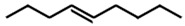	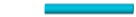
Dienes	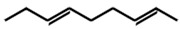	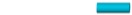
Trimethylalkanes	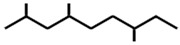	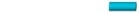
Methylalkenes	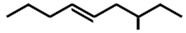	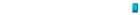
Tetramethylalkanes	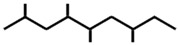	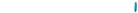
Trienes	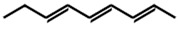	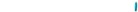
Dimethylalkenes	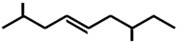	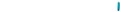
–	–	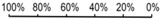

**Table 2:** Major classes of plant wax compounds, adapted form [69,96,98]. Classes of compounds also known to occur in ant cuticle are reported in bold [2,61,62].

Compound Classes	Chemical Structure	Chain Lenght
***n*-Alkanes**	CH_3_(CH_2_)*_n_*CH_3_	[C_21_–C_35_]
Secondary alcohols	CH_3_(CH_2_)*_n_*CHOH(CH_2_)*_m_*CH_3_	[C_21_–C_35_]
**Ketones**	CH_3_(CH_2_)*_n_*CO(CH_2_)*_m_*CH_3_	[C_21_–C_35_]
**Fatty alcohols**	CH_3_(CH_2_)*_n_*CH_2_OH	[C_22_–C_34_]
**Fatty acids**	CH_3_(CH_2_)*_n_*CO_2_H	[C_16_–C_34_]
**Aldehydes**	CH_3_(CH_2_)*_n_*CHO	[C_21_–C_35_]
**Wax esters**	CH_3_(CH_2_)*_n_*CO_2_(CH_2_)*_m_*CH_3_	[C_32_–C_64_]
Diketones	CH_3_(CH_2_)*_n_*COCH_2_CO(CH_2_)*_m_*CH_3_	[C_27_–C_33_]
Triterpenoids	C_30_H_50_O	See [97]
